# E-Selectin Is Associated with Daytime and 24-Hour Diastolic Blood Pressure Variability in Type 2 Diabetes

**DOI:** 10.3390/biomedicines10020279

**Published:** 2022-01-26

**Authors:** Dana Mihaela Ciobanu, Cornelia Bala, Adriana Rusu, Gabriel Cismaru, Gabriela Roman

**Affiliations:** 1 Diabetes and Nutrition Diseases, Department 6 Medical Specialties, Faculty of Medicine, Iuliu Hațieganu University of Medicine and Pharmacy, 400006 Cluj-Napoca, Romania; dana.ciobanu@umfcluj.ro (D.M.C.); cbala@umfcluj.ro (C.B.); groman@umfcluj.ro (G.R.); 2 Cardiology-Rehabilitation, Department 5 Internal Medicine, Faculty of Medicine, Iuliu Hațieganu University of Medicine and Pharmacy, 400437 Cluj-Napoca, Romania; cismaru.gabriel@umfcluj.ro

**Keywords:** E-selectin, blood pressure variability, 24 h ambulatory blood pressure monitoring, type 2 diabetes mellitus

## Abstract

E-selectin is an endothelial cell adhesion molecule involved in vascular inflammation. Elevated E-selectin has been reported in patients with high blood pressure and diabetes. Given the increasing clinical relevance of parameters derived from ambulatory blood pressure monitoring, further investigation of their relationships with E-selectin is of interest. In this study, we aimed to investigate the association between serum E-selectin, office blood pressure and 24 h ambulatory blood pressure parameters in patients with type 2 diabetes. Blood pressure variability was assessed by computing the standard deviation of mean systolic and diastolic blood pressure separately for daytime and nighttime during 24 h ambulatory blood pressure monitoring in a cohort of patients with type 2 diabetes (*n* = 132). Additionally, were assessed nighttime systolic dipping and pulse pressure separately for daytime, nighttime, and 24 h. Serum E-selectin was measured using the enzyme-linked immunosorbent assay technique. We found that E-selectin was consistently associated with 24 h diastolic blood pressure variability (r = 0.238; *p* = 0.019) and daytime diastolic blood pressure variability (r = 0.258; *p* = 0.012), after adjustment for confounding factors. No association of E-selectin with office blood pressure and other 24 h ambulatory blood pressure parameters was observed. In conclusion, endothelial activation indicated by elevated serum E-selectin is associated with increased ambulatory diastolic blood pressure variability in patients with type 2 diabetes.

## 1. Introduction

The investigation of inflammatory mechanisms of high blood pressure (BP) has gained increased interest in recent decades. Numerous cytokines regulate vascular tone and maintain the appropriate balance between pro- and anti-inflammatory factors involved in hypertension and vascular disease [1]. Existing evidence suggest that a chronic proinflammatory state accompanies hypertension [2], and that inflammation may be involved in both hypertension development and end-organ damage [3]. The mechanisms involved in the link between inflammation and hypertension is represented by excessive production of reactive oxygen species with consequent release of proinflammatory cytokines (like tumor necrosis factor [TNF]-α, interleukin [IL]-6, IL-17), increased expression of intracellular adhesion molecule (ICAM)-1 and vascular cell adhesion molecule (VCAM)-1 at endothelial level, decreased nitric oxide (NO) formation and endothelial dysfunction [1,4]. 

Selectins are a family of endothelial leukocyte cell adhesion molecules involved in vascular disease progression by causing a local inflammatory response through stimulation of the migration of immune cells across the endothelial barriers [5,6]. The endothelial-specific molecule E-selectin appears to be involved in worsening the cardiometabolic risk profile since its circulating levels were shown to be associated with gradual deterioration of two traditional risk factors, BP and glycemia [7,8,9]. E-selectin was reported to be involved in the pathogenesis of type 2 diabetes [10,11] and hypertension [12,13] by influencing the inflammatory process associated with microvascular endothelial dysfunction. Increased blood concentration of pro-inflammatory E-selectin was found in patients with essential hypertension compared to their normotensive peers and it was explained by the hemodynamic load caused by high BP that resulted in changes in vascular structure and reduced dilation capacity of blood vessels [12]. Endothelial inflammatory activation suggested by high E-selectin levels in hypertensive patients [13] were consistent with findings suggesting increased expression of the E-selectin gene in relation to the risk of hypertension development [14].

Recently, increased short-term BP variability within 24 h has been associated with a higher incidence of cardiovascular events, increased risk of end-organ damage, and increased all-cause mortality in patients with hypertension, independent of average systolic and diastolic BP values [15,16,17]. Sustained increases in BP variability are caused by abnormal function of homeostatic cardiovascular regulatory mechanisms or may be due to associated pathological conditions (increased sympathetic tonus during the night, sleep-disordered breathing, obesity, insulin resistance) [16]. In the presence of both type 2 diabetes and hypertension, higher ambulatory BP variability was reported compared to the presence of hypertension alone [17]. 

Typically, BP has a circadian variation with the lowest levels observed during sleep [18]. An arbitrary cutoff of >10% has been selected and is currently used for defining this normal decrease in the nighttime BP as compared to the daytime. The absence of nocturnal BP dipping was associated with obesity, obstructive sleep apnea, increased salt intake, chronic kidney disease, autonomic failure, and old age [19]. Additionally, non-dipping status is frequent among patients with diabetes [20] and has been linked to the presence of autonomic neuropathy [21], increased sympathetic activation due to insulin resistance [22,23], and increased sodium reabsorption [24]. Non-dipping systolic BP has been associated with increased cardiovascular morbidity and mortality independent of BP values in both the general population and patients with diabetes [25,26,27]. Additionally, in patients with diabetes, non-dipping status has been associated with an increased risk for microalbuminuria and diabetic nephropathy [28,29].

Pulse pressure represents the difference between the systolic and diastolic BP and results from the interaction of arterial stiffness (increased due to atherosclerosis or age) and cardiac ejection [30]. Increased pulse pressure is frequent among patients with diabetes, is considered as the clinical expression of arterial stiffness [31], and represents a significant predictor of cardiovascular, especially coronary, disease in patients with hypertension and those with diabetes [31,32]. 

Vascular inflammation indicated by elevated E-selectin levels was associated with increased office BP [8], mean BP [33], and awake systolic BP variability [34] in the general population. However, data on the relationship between E-selectin levels and parameters evaluated 24 h ambulatory BP monitoring in persons with diabetes are missing. In this context, we aimed to investigate the association between serum E-selectin, office BP and 24 h ambulatory BP parameters (mean BP, BP variability, nighttime systolic dipping, dipping status, and pulse pressure) in patients with type 2 diabetes.

## 2. Materials and Methods

### 2.1. Study Design and Patients

This was a cross-sectional study that included data from adult patients with type 2 diabetes (*n* = 132) presenting at Emergency Clinical County Hospital, Department of Diabetes and Nutrition in Cluj-Napoca, Romania. The diagnosis of type 2 diabetes and its chronic micro- and macrovascular complications (retinopathy, polyneuropathy, chronic kidney disease, atherosclerotic cardiovascular disease) was established according to the American Diabetes Association criteria [35]. Hypertension was diagnosed if the office BP was ≥140 mmHg systolic or ≥90 mmHg diastolic or the patient was using antihypertensive drugs [35]. Patients were not included in the study if they had any of the following conditions: unstable cardiovascular conditions, secondary hypertension, liver and renal failure, inflammatory diseases, malignancies, pregnancy, breastfeeding.

The study was conducted according to the International Conference on Harmonization’s Good Clinical Practice Guidelines and the Declaration of Helsinki. The study protocol and informed consent were approved by the Ethics Committee of the “Iuliu Hatieganu” University of Medicine and Pharmacy Cluj-Napoca, Romania (approval number 868/25.09.2013). All participants provided written informed consent before any study procedure.

### 2.2. Study Protocol

Age, sex, height, weight, abdominal circumference, smoking status, duration of diabetes, duration of hypertension, as well as chronic complications of diabetes were collected by patient interviews, physical examination, and from patients’ medical files. Body mass index was calculated as $\frac{weight\;\left(kg\right)}{height\;\left(m^{2}\right)}\;$. Office BP was measured in a sitting position after 5 min of rest. Fasting blood samples were obtained from each patient for the assessment of glycemia, glycated hemoglobin (HbA1c), lipid profile (total cholesterol, triglycerides, HDL cholesterol), creatinine, and E-selectin. Serum obtained after blood centrifugation (4500 rpm, 10 min) was stored until E-selectin analysis at −80 °C. Serum levels of E-selectin were quantified using a commercially available enzyme-linked immunosorbent assay (ELISA) human kit according to the manufacturers’ instructions (Wuhan EIAab Science, Wuhan, China, Catalog No: E0029h) at Laboratory of Immunology of the Emergency Clinical County Hospital Cluj-Napoca, Romania. The detection limit of the used assay ranged between 0.156 and 10 ng/mL. Serum E-selectin level was calculated using the reference to standard curves concentrations. The sensitivity of the ELISA system was less than 0.016 ng/mL. The inter-assay coefficient of variance was less than 12% and the intra-assay coefficient of variance was less than 10%. 

Biochemical laboratory investigations and HbA1c were assessed on the day of blood collection using commercially available methods at Central Laboratory of the Emergency Clinical County Hospital Cluj, Romania (Beckman Coulter AU480 Chemistry Analyzer, Brea, CA, USA). 

### 2.3. 24 h Ambulatory Blood Pressure Monitoring and Parameters Assessed

After blood collection, all study participants underwent 24 h ambulatory BP monitoring using a verified automatic device based on the oscillometric method, HolCARD CR-07 (Aspel, Zabierzów, Poland). The BP measurement was performed at the arm with the highest BP and values were recorded every 30 min during daytime and every 60 min during nighttime. Patients were permitted to engage in their normal daily activities during the 24 h ambulatory BP monitoring evaluation, but to refrain from intense physical activity and to keep their arm still and relaxed during BP measurements [36]. All patients enrolled had complete data on at least 70% of all possible measurements for daytime and covering nighttime period too. Standard deviation (SD) of mean systolic and diastolic BP was calculated to evaluate short-term BP variability during the daytime, nighttime and 24 h periods [37]. Nocturnal systolic BP dipping was calculated as $100*\left(1-\frac{mean\;nighttime\;systolic\;BP}{mean\;daytime\;systolic\;BP}\right)$. Participants were classified as dippers if the nocturnal systolic BP fall was ≥10% and non-dippers if it was <10% (defined as higher nighttime mean systolic BP than daytime mean systolic BP) [38,39]. Pulse pressure during the daytime, nighttime and 24 h periods was computed as the difference between mean systolic BP and mean diastolic BP for the corresponding period [40]. 

### 2.4. Statistical Analysis

Descriptive statistics used were number and percentages, mean ± standard deviation, median or percentile (25th and 75th) after assessing the normal distribution using the Shapiro–Wilk test. BP and BP variability parameters were analyzed for 24 h and separately for daytime and nighttime. Daytime BP consisted of all BP values recorded between 7:00 and 22:00; nighttime BP consisted of all BP values recorded between 22:00 and 7:00. For daytime BP measurements, we calculated individual means for each hour. These means were further used to compute mean systolic and diastolic BP during daytime as the average of the computed means. The 24 h mean systolic and diastolic BP were computed as the average of computed means during daytime and hourly recorded data for nighttime [41]. The variability of BP was assessed by computing the standard deviation (SD) of mean systolic and diastolic BP separately for daytime and nighttime. For these time intervals the SD was calculated as $\sqrt{\frac{1}{N-1}\overset{N}{\underset{k=1}{\sum }}\left|BP_{k+1}-\bar{BP}\right|}$, where *N* is the number of valid *BP* measurements in a participant, *BP_k_* represents the *BP* value at moment *k* and $\bar{BP}$ is the mean *BP* for the time assessed [42]. To account for the circadian variation of *BP*, its variability for 24 h was computed as the weighted mean of daytime and nighttime SD of *BP* [42,43].

Univariate linear regression analysis unadjusted and adjusted for age, sex, smoking status, body mass index, duration of diabetes and hypertension, fasting glycemia, and HbA1c was performed to assess the association of E-selectin (as dependent variable) with office BP and 24 h ambulatory BP parameters (BP mean, BP variability, nocturnal dipping, dipper status, and pulse pressure). E-selectin had a non-Gaussian distribution and therefore was logarithmically transformed (lgE-selectin) before inclusion in the regression analysis. Collinearity diagnostic was performed for BP mean, BP variability parameters, nocturnal dipping, dipper status, and pulse pressure before linear regression analysis. Several variables included as predictors displayed variation inflation factors >10 when included in the multivariate regression, indicating potential collinearity issues among predictors. Thus, it was decided not to perform multivariate regression analysis with office BP and 24 h ambulatory BP parameters included as predictors in a single model. Statistical analyses were performed using IBM SPSS Statistics for Windows, Version 22.0 (IBM Corp, Armonk, NY, USA). The statistical significance threshold was considered *p* < 0.05.

## 3. Results

### 3.1. Characteristics of the Study Group

Of the whole group, 45.5% were men and 12.1% were current smokers. Participants had a mean age of 60.1 years, body mass index 32.6 kg/m^2^, fasting glycemia 156.5 mg/dL, a median HbA1c 9.2% and a median diabetes duration 8.0 years. Hypertension was previously diagnosed and treated with medication in 88.6% of all patients and had a median duration of 8.0 years. Median E-selectin levels were 2.5 ng/mL (Table 1).

The 24 h ambulatory BP monitoring parameters are summarized in Table 2. The mean daytime systolic BP was 130.8 mmHg and mean nighttime systolic BP 125.0 mmHg, with similar variability (SD 3.0). The mean daytime diastolic BP was 82.6 mmHg and mean nighttime diastolic BP was 72.0 mmHg, with variability of 2.8 during the day and 2.7 during the night. The mean value of nocturnal systolic dipping was 5.3% and 73.5% of the patients were non-dippers.

### 3.2. Association of E-Selectin with Office Blood Pressure

**Figure 1:**
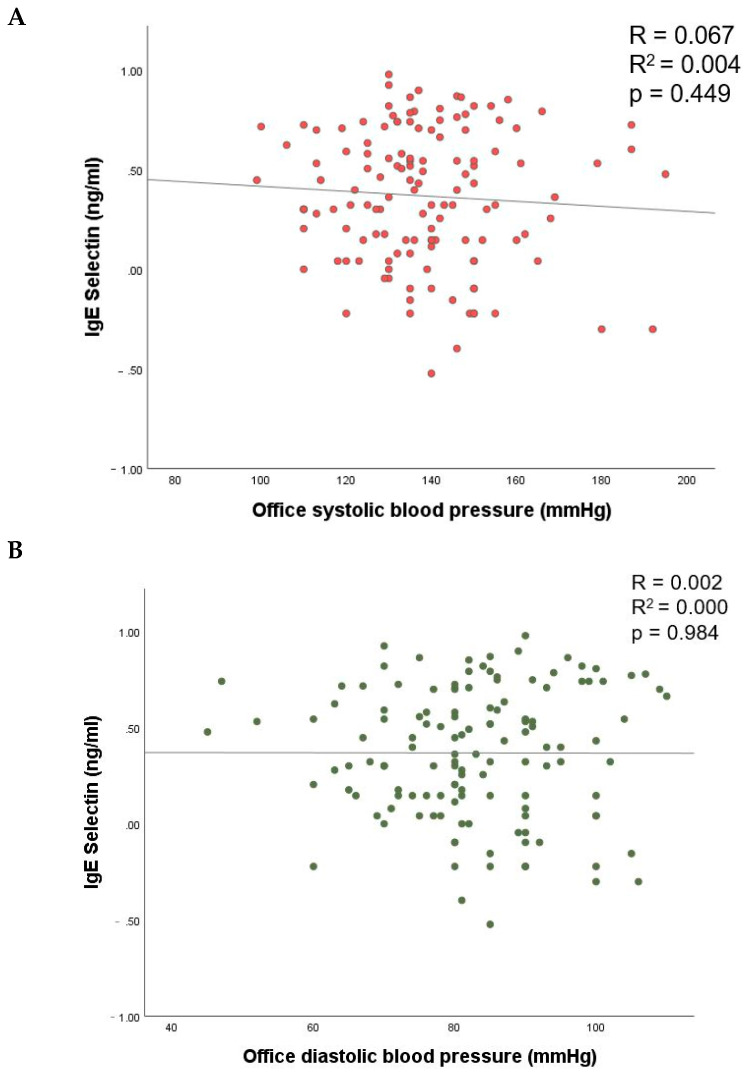
The relationship of E-selectin with office systolic (**A**) and diastolic (**B**) blood pressure.

No association of lgE-selectin with office systolic and diastolic parameters was observed neither in the unadjusted nor in the adjusted model (*p* > 0.05 for all; Figure 1, Table 3).

### 3.3. Association of E-Selectin with 24 h Ambulatory Blood Pressure Parameters

**Figure 2:**
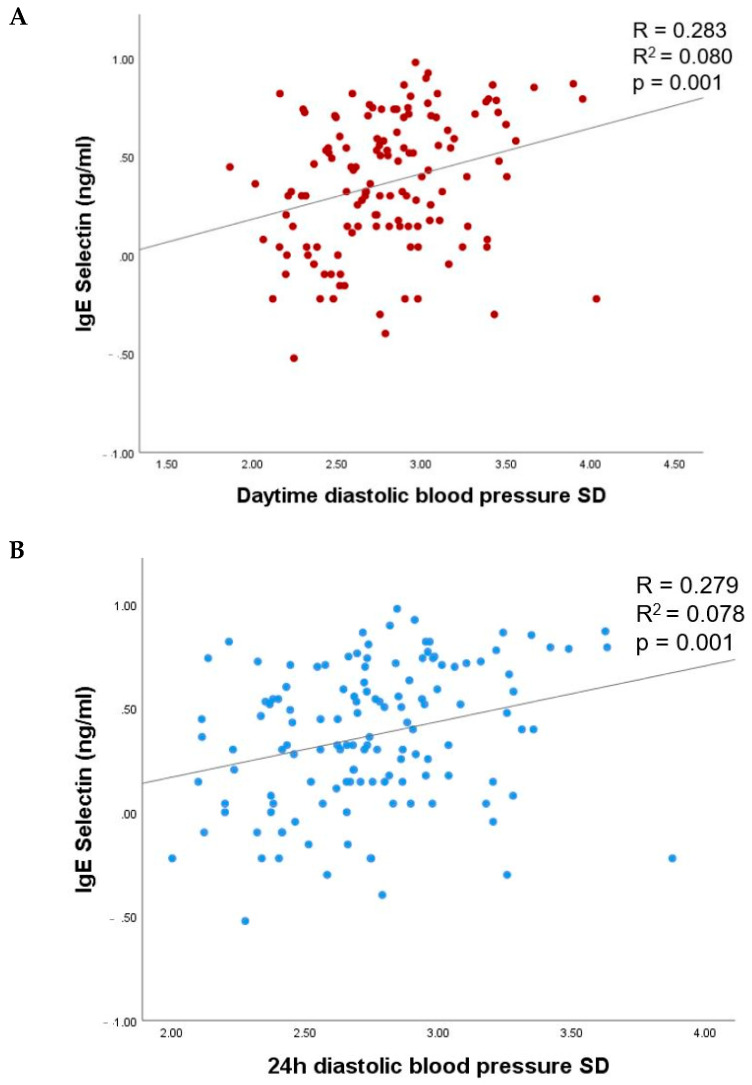
The relationship of lgE-selectin with daytime (**A**) and 24 h (**B**) BP variability. SD, standard deviation.

Of the 24 h ambulatory BP parameters, lgE-selectin was significantly associated with daytime and 24 h diastolic BP variability in unadjusted linear regression analysis (*p* values < 0.001; Figure 2, Table 4). No association of lgE-selectin with systolic daytime, nighttime or 24 h BP variability, mean systolic or diastolic BP, nighttime systolic dipping, dipping status, or pulse pressure was observed in the unadjusted linear regression (*p* > 0.05 for all).

In linear regression analysis adjusted for age, sex, smoking status, fasting glycemia, HbA1c, body mass index, diabetes, and hypertension duration, lgE-selectin remained significantly associated with daytime and 24 h diastolic BP variability in patients with type 2 diabetes (β = 0.258, *p* = 0.012 and β = 0.238; *p* = 0.019, respectively). No association was observed with other 24 h BP parameters assessed (Table 4).

## 4. Discussion

In the present study, we addressed the issue of E-selectin levels in hypertension and diabetes and sought to understand its relationship with parameters evaluated during 24 h ambulatory BP monitoring. We found that elevated serum E-selectin was associated with daytime and 24 h diastolic BP variability in patients with type 2 diabetes. Previous studies described the role of E-selectin in the inflammation and activation of vascular endothelial cells in response to certain pro-inflammatory stimuli [9,10,11,12,13,33,34]. Elevated E-selectin levels were previously reported to be positively correlated with daytime systolic BP variability and not with daytime diastolic BP variability in patients with newly diagnosed hypertension without diabetes [34]. Additionally, recent data published from the Maastricht Study in which 28% of the enrolled participants had type 2 diabetes indicated no associations between short-term BP variability and a composite score of plasma biomarkers that included E-selectin [44]. The difference between these previous reports and our results could be explained by the inclusion in our study of only patients with type 2 diabetes. Our observation suggests that the constant elevation of diastolic BP variability evaluated during 24 h ambulatory BP monitoring can trigger the expression of E-selectin in the endothelial cells, independent of confounding factors known to increase E-selectin levels: age, sex, body mass index, glycemia, duration of diabetes, and hypertension [10,12,45]. In addition, it is noteworthy that our results suggest that increased diastolic BP variability might play a pathogenic role in endothelial dysfunction by increasing E-selectin. Cellular adhesion molecules, such E-selectin, play an important role in the initiation of atherosclerosis [46]. E-selectin is found on the surface of endothelial cells and recruits circulatory leukocytes, promoting their adhesion to endothelium [46]. Increased E-selectin levels have been described in proinflammatory states (obesity and type 2 diabetes) [47,48] and in hypertensive patients [12]. In this later group, E-selectin was associated with endothelial dysfunction and vascular structural changes [12] and it has been hypothesized that increased E-selectin expression and cytokine release secondary to leukocytes recruitment and accumulation may represent a mechanism through which inflammation may induce vascular changes [12,49]. 

Hypertension is found at higher rates in people with diabetes and it represents a clear risk factor for cardiovascular disease, while diabetes itself also confers an independent risk [21]. BP variability is elevated in hypertension and it increases with the severity of hypertension [15]. Although mean BP levels are largely considered to be associated with adverse cardiovascular outcomes, evidence also suggests a possible role of increased BP variability in this regard [15]. Increased nighttime systolic and diastolic BP variability resulted in a greater risk of cardiovascular events and was able to stratify patients with hypertension among risk categories [50]. In patients with diabetes, overall BP variability assessed using 24 h ambulatory BP monitoring was reported to be frequently increased compared to patients with hypertension and normal glycemia [17]. This evidence suggests that ambulatory BP monitoring might be a powerful tool for better identification and stratification of cardiovascular risk related to increased mean BP and BP variability in patients with type 2 diabetes. Although E-selectin is on the average higher in patients with hypertension [12,14] and diabetes [10,11], the interest for routine E-selectin measurement in the future will depend on the information it will yield in stratifying patients for diagnostic and prognostic purposes. Ongoing clinical studies are aiming to investigate the influence of BP variability on serum E-selectin [44]. Given the relationship we described between BP variability and E-selectin, it may be possible that increased serum E-selectin levels might be detected before adverse cardiovascular outcomes become clinically manifest. 

Epidemiological data show a linear association between elevated office BP and risk of acute cardiovascular events [40]. Given the relation of E-selectin with markers of the atherogenic process [9,51] and cardiovascular events [10], we investigated the association of E-selectin with office BP, and we found no association between these parameters. Our results are in line with two previous studies that showed no significant association of E-selectin with office BP in patients with newly diagnosed diabetes and children and adolescents with primary hypertension [52]. However, recently, Lee et al. reported a significant and positive association of E-selectin with both systolic and diastolic office BP in women from the general population, but not in men [53]. A possible explanation of the difference between this latter study and our results may be the analysis of both genders in our study as opposed to separate analysis for men and women in the study of Lee et al. [53]. It is known that selectins are involved in female reproduction by regulation of ovarian function and menopause [54] and this may explain the association of E-selectin with office BP reported in women [53]. Another explanation for our results is the white-coat effect which may have influenced office BP measurement [55]. However, the white-coat effect is not present in the 24 h BP monitoring and still, we found no significant association of E-selectin with mean systolic and diastolic BP measured during the daytime, nighttime and 24 h periods. Conversely, in a study evaluating newly diagnosed patients with hypertension (none of them with diabetes) mean systolic BP was associated with E-selectin in multivariate analysis [34]. 

We did not find a significant association of E-selectin with pulse pressure during daytime, nighttime and 24 h periods. High values of pulse pressure indicate elevated arterial stiffness, a feature frequently found in persons with diabetes [56] and hypertension [57]. However, the physiological mechanisms explaining the increase in pulse pressure in persons with diabetes are yet to be identified. Vascular adhesion cells were previously evaluated as putative determinants of changes in pulse pressure levels. Among them, E-selectin showed no significant association with pulse pressure in patients with type 1 diabetes during 20-year follow-up [58] nor in a large cohort of Mongolian residents [59]. Ferreira et al. reported that E-selectin decreased over time despite an increase in BP levels and higher prevalence and incidence of hypertension in the participants with type 1 diabetes followed for 20 years, while other adhesion molecules had an ascending trajectory [58]. In both studies, pulse pressure was calculated based on office BP values [58,59]. It has been shown that 24 h ambulatory BP monitoring is a better predictor of hypertension-mediated organ damage than office BP, although BP values are lower on average. Consistent evidence points to a higher sensitivity of cardiovascular risk prediction when BP is monitored continuously for 24 h [39]. Although arterial stiffness plays an important role in atherosclerotic cardiovascular disease, its relationship with E-selectin could not be proven. An interesting hypothesis is that endothelial dysfunction indicated by elevated E-selectin might develop earlier than arterial stiffness [60] and this may explain the lack of association of E-selectin with pulse pressure. 

Persons presenting with reduced BP fall during nighttime, defined as non-dipper, have been proven to have an increased cardiovascular risk [25] and a large proportion of patients with type 2 diabetes included in our study were non-dipper. Reverse dipper pattern (dipping index >20%) and non-dipper pattern have been independently associated with type 2 diabetes in hypertensive patients [61]. Furthermore, the reverse dipper pattern was associated with a higher risk of type 2 diabetes when compared to dipper and non-dipper patterns [61]. Still, we could not find a significant association of dipping patterns with E-selectin. The time of day when antihypertensive drugs are taken plays a significant role in nocturnal BP fall [62] and this might have influenced our results. Additionally, another explanation is the use of certain antihypertensive drugs such as long-acting calcium channel blockers and angiotensin II-receptor blockers which have been shown to decrease E-selectin levels [63,64]. 

Our study investigating E-selectin associations with parameters evaluated during 24 h ambulatory BP monitoring brings new and relevant data supporting the involvement of endothelial dysfunction and inflammation in worsening of cardiovascular risk. In addition, these results are representative for patients with type 2 diabetes presenting with multiple comorbidities and chronic complications of diabetes, in comparison with previously mentioned studies enrolling persons from the general population, with metabolic syndrome or hypertension only. Current therapeutic strategies targeting hypertension, hyperglycemia, or hypercholesterolemia that improve atherosclerotic cardiovascular diseases outcomes are available. Data is also accumulating regarding their role in lowering proinflammatory cytokines and endothelial dysfunction [1,65]. However, future development of targeted E-selectin therapies aiming to treat vascular inflammation and further inhibit atherosclerosis progression [66] may further improve the cardiovascular outcomes of patients with or without diabetes.

Our observational study suggests that E-selectin is associated with diastolic BP variability, but the causality could not be assessed due to its cross-sectional design. Another limitation of this study is the arbitrary definition of night and day, similarly to those previously reported in other studies [34,36,44]. We did not use any diaries or actigraphs to document activity/rest cycles. A prospective study with a larger sample is warranted to ascertain if the increase in serum E-selectin levels could reflect endothelial cell damage caused by increased diastolic BP variability or, if E-selectin could be itself a determinant of increased diastolic BP variability. Thought, our exploratory observation suggests that serum E-selectin might be implicated in the endothelial activation associated with hypertension and diabetes.

## 5. Conclusions

The association of serum E-selectin with daytime and 24 h diastolic BP variability indicates that endothelial activation might be linked to increased BP variability in patients with type 2 diabetes. Increased short-term BP variability might stimulate vascular inflammation indicated by high E-Selectin levels, which may, in turn, determine pathological changes in the arterial wall. 

## Figures and Tables

**Table 1:** Clinical and laboratory characteristics of the study sample.

Parameters	Patients (N = 132)
Men, n (%)	60 (45.5%)
Age, years	60.1 ± 8.0
Smokers, n (%)	16 (12.1%)
Waist circumference, cm	111.0 ± 11.6
Body mass index, kg/m^2^	32.6 ± 5.3
Total cholesterol, mg/dL	197.6 ± 45.7
HDL cholesterol, mg/dL	43.0 (38.0; 51.5)
Tryglicerides, mg/dL	189.0 (124.0; 248.0)
Creatinine, mg/dL	0.9 (0.8; 1.1)
Diabetes duration, years	8.0 (3.0; 13.5)
HbA1c, %	9.2 (8.2; 11.2)
Fasting glycemia, mg/dL	156.5 (132.0; 197.0)
Diabetic retinopathy, n (%)	41 (31.1%)
Diabetic polineuropathy, n (%)	76 (57.6%)
Chronic kidney disease, n (%)	46 (34.8%)
Atherosclerotic cardiovascular disease, n (%)	55 (41.7%)
Hypertension, n (%)	117 (88.6%)
Hypertension duration, years	8.0 (4.0; 13.0)
Office systolic blood pressure, mmHg	136.5 (128.5; 148.5)
Office diastolic blood pressure, mmHg	82.6 ± 12.5
E-selectin, ng/mL	2.5 (1.4; 4.8)

N/n, number; %, percentage; HbA1c, glycated hemoglobin.

**Table 2:** 24 h ambulatory blood pressure parameters.

Blood Pressure (mmHg)	Patients (N = 132)
Systolic Mean, mmHg	
Daytime	130.8 (123.2; 141.0)
Nighttime	125.0 ± 15.8
24 h	122.1 (115.1; 130.0)
Systolic Variability, mmHg	
Daytime	3.0 ± 0.6
Nighttime	3.0 ± 0.6
24 h	3.0 ± 0.5
Diastolic Mean, mmHg	
Daytime	82.6 ± 10.8
Nighttime	72.0 (64.7; 79.1)
24 h	78.0 ± 7.8
Diastolic Variability, mmHg	
Daytime	2.8 ± 0.4
Nighttime	2.7 ± 0.5
24 h	2.7 ± 0.4
Nocturnal Systolic Dipping, %	5.3 ± 6.1
Non-dippers, n (%)	97 (73.5%)
Pulse pressure, mmHg	
Daytime	49.5 (42.9; 60.1)
Nighttime	49.8 (41.2; 60.8)
24 h	49.7 (43.1; 60.0)

n/N, number; %, percentage.

**Table 3:** Univariate linear regression analysis for the dependent variable lgE-Selectin using office blood pressure parameters as predictors.

Blood Pressure (mmHg)	LgE-Selectin	
	Beta Standardized Coefficients	*p*-Value
Unadjusted regression		
Office systolic blood pressure	−0.067	0.449
Office diastolic blood pressure	−0.002	0.984
Adjusted regression *		
Office systolic blood pressure	−0.164	0.107
Office diastolic blood pressure	−0.102	0.312

* Model adjusted for age, sex, smoking status, fasting glycemia and HbA1c, diabetes and hyper-tension duration, body mass index.

**Table 4:** Univariate linear regression analysis for the dependent variable lgE-Selectin using 24 h ambulatory blood pressure parameters as predictors.

Blood Pressure (mmHg)	LgE-Selectin	*p*-Value
	Beta Standardized Coefficients	
Unadjusted regression		
Systolic Variability		
Daytime	0.119	0.174
Nighttime	0.115	0.190
24 h	0.141	0.106
Diastolic Variability		
Daytime	0.283	0.001
Nighttime	0.087	0.321
24 h	0.279	0.001
Systolic Mean		
Daytime	0.115	0.189
Nighttime	0.147	0.092
24 h	0.124	0.157
Diastolic Mean		
Daytime	0.046	0.604
Nighttime	0.034	0.699
24 h	−0.012	0.890
Nocturnal Systolic Dipping	0.080	0.364
Systolic Dipping Status	−0.064	0.468
Pulse Pressure		
Daytime	0.099	0.257
Nighttime	0.137	0.118
24 h	0.112	0.203
Adjusted regression *		
Systolic Variability		
Daytime	0.093	0.364
Nighttime	0.126	0.181
24 h	0.133	0.190
Diastolic Variability		
Daytime	0.258	0.012
Nighttime	0.043	0.656
24 h	0.238	0.019
Systolic Mean		
Daytime	0.078	0.439
Nighttime	0.134	0.170
24 h	0.098	0.323
Diastolic Mean		
Daytime	−0.013	0.896
Nighttime	0.011	0.915
24 h	−0.022	0.822
Nocturnal Systolic Dipping Index	−0.109	0.236
Systolic Dipping Status	−0.055	0.559
Pulse Pressure		
Daytime	0.137	0.227
Nighttime	0.183	0.101
24 h	0.154	0.175

* Model adjusted for age, sex, smoking status, fasting glycemia and HbA1c, diabetes and hypertension duration, body mass index.

## Data Availability

The data presented in this study are available on request from the corresponding author. The data are not publicly available as written consent was not obtained from study participants for this.
